# Network topological alterations tied to plasma pathology markers in unimpaired individuals

**DOI:** 10.1002/alz70856_096512

**Published:** 2025-12-24

**Authors:** Alejandra García‐Colomo, David López‐Sanz, Martín Carrasco‐Gómez, Cornelis J. Stam, Arjan Hillebrand, Fernando Maestú

**Affiliations:** ^1^ Universidad Complutense de Madrid, Madrid, Madrid, Spain; ^2^ Complutense University of Madrid, Madrid, Madrid, Spain; ^3^ Universidad Politécnica de Madrid, Madrid, Madrid, Spain; ^4^ Clinical neurophysiology and MEG Center, Amsterdam University Medical Center, location VUmc, Amsterdam, Noord‐Holland, Netherlands; ^5^ Amsterdam UMC location Vrije Universiteit Amsterdam, Department of Clinical Neurophysiology and MEG Center, Neurology, De Boelelaan 1117, Amsterdam, Netherlands

## Abstract

**Background:**

The preclinical stage of Alzheimer's disease (AD) has gained attention for the window of opportunity it opens for early detection and intervention. As a result, electrophysiology and plasma biomarkers are emerging as great candidates in this pursuit due to their low invasiveness.

This is the first magnetoencephalography study to assess the relationship between network topology parameters, calculated from magnetoencephalography (MEG) recordings using minimum spanning tree (MST), and plasma phosphorylated tau231 (ptau231) levels in unimpaired individuals, with different risk levels of Alzheimer's disease.

**Method:**

The sample consisted of 76 individuals with available MEG recordings and *p*‐tau231 concentrations. The MST for the theta, alpha and beta bands for each subject was obtained, and the leaf fraction, tree hierarchy and diameter were calculated. The association between these measures was analysed for the whole sample, and for each of the risk subgroups.

**Result:**

Increasing concentrations of *p*‐tau231 were associated with greater leaf fraction and tree hierarchy values, along with lower diameter values, for the alpha and theta frequency bands. These results emerged for the whole sample and the higher risk group, but not for the lower risk group.

**Conclusion:**

Our results indicate that the network topology of cognitively unimpaired individuals with elevated plasma phosphorylated tau231 levels, a marker of Alzheimer's disease pathology and amyloid‐β accumulation, is already altered, shifting towards a more integrated network increasing its vulnerability and hub‐dependency, mostly in the alpha band. This is indicated by increases in leaf fraction and tree hierarchy, along with reductions in diameter. These results match the initial trajectory proposed by theoretical models of disease progression and network disruption and suggest that changes in brain function and organisation begin early on.

